# High Dynamic Range CsFAPbI_3_ Perovskite Photodetectors with 12.7 MHz Bandwidth

**DOI:** 10.3390/ma19071315

**Published:** 2026-03-26

**Authors:** Abdul Mannan Majeed, Sandra Stanionytė, Gediminas Kreiza, Patrik Ščajev

**Affiliations:** 1 Institute of Photonics and Nanotechnology, Faculty of Physics, Vilnius University, Sauletekio Ave. 3, LT-10257 Vilnius, Lithuania; gediminas.kreiza@ff.vu.lt; 2 Center for Physical Sciences and Technology, Sauletekio Ave. 3, LT-10257 Vilnius, Lithuania; sandra.stanionyte@ftmc.lt

**Keywords:** perovskite photodetectors, responsivity, bandwidth

## Abstract

We develop Cs_x_FA_1−x_PbI_3_ perovskite photodetectors with varying Cs content in the x = 0.05–0.25 range to identify the most stable cubic-lattice perovskite composition for visible-light photodetection. The perovskite layers were deposited by the spin-coating technique on a nickel oxide p-type contact and then were covered with C_60_/Ag electron contact to obtain a vertical pin diode structure. X-ray diffraction (XRD) and scanning electron microscopy (SEM) measurements show that x = 0.1–0.2 provides the most stable lattice and pinhole-free perovskite layers. The photocurrents are linear in an extremely wide 1 nW–10 mW excitation power range, providing photoresponsivity of 0.28 A/W at 532 nm (green light), similar to that of Si photodiodes. The testing of the photodetectors using picosecond pulses provided their rise times and fall times. The x = 0.2 composition provided the shortest rise time values of 27.5 ns, leading to a detector modulation bandwidth of 12.7 MHz. This indicates that this perovskite composition is suitable for replacing silicon photodetectors in cost-efficient light detection systems for imaging and light communication applications such as Li-Fi.

## 1. Introduction

The term “perovskite” originated from the mineral calcium titanate (CaTiO_3_), first identified by Gustav Rose in 1839 [1], in the Ural Mountains. The mineral was later named in honor of the mineralogist L.A. Perovski, who made significant contributions to the study of its structure. Over time, the definition of perovskite has broadened to encompass all materials that share the same crystal structure as CaTiO_3_. Today, perovskite materials are regarded as highly promising for a broad range of optoelectronic applications due to their exceptional properties, including a high absorption coefficient, high defect tolerance, long carrier lifetime, long diffusion length, and high charge-carrier mobility [2,3,4,5,6]. Since the first report in 2009 [7], the efficiency of perovskite-based solar cells has improved dramatically, increasing from an initial 3.8% to values exceeding 25% [8,9,10]. In addition to photovoltaic devices, perovskites are widely used in the production of photodetectors [11,12,13,14,15], X-ray detectors [16], lasers [17,18], light-emitting diodes [19,20], etc.

Photodetectors (PDs) play a vital role in imaging [21], communication [3], and medical diagnostics [22,23] technologies by converting incident light into electrical signals. Based on their spectral responsivity bandwidth, they are typically divided into wideband and narrowband photodetectors. Wideband devices provide uniform spectral responses with high external quantum efficiency (EQE) over a wide wavelength range, while narrowband devices are tailored for selective detection of specific spectral regions, including individual visible colors or defined infrared bands [24]. Organic-inorganic lead-based perovskites are suitable for the construction of efficient photodetectors due to their excellent optoelectronic properties, with a low-cost fabrication process [13,14,25]. On the other hand, despite their high efficiency and fast response, conventional silicon or gallium arsenide-based photodetectors are often restricted by high-cost fabrication processes [26,27].

Cost-effective, solution-processed perovskite photodetectors offer a cost-effective alternative to conventional thick silicon detectors [28]. The estimated areal cost of used Cs_0.2_Fa_0.8_PbI_3_ perovskite material is 6 × 10^−6^ Eur/m^2^ [29,30], while Si and GaAs prices are 2.5 × 10^−5^ Eur/m^2^ [31] and 1.4 × 10^−4^ Eur/m^2^ [32], respectively (typically Si and GaAs ultrafast photodiode thicknesses are 10 μm and 500 nm, respectively; the thickness for silicon is much larger, as it is an indirect-bandgap semiconductor).

The intrinsic properties of halide perovskites open new opportunities for advanced sensing technologies. Their soft lattice structure and low-temperature processability enable direct deposition on a wide variety of substrates, making them particularly suitable for low-light-level detection and Li-Fi communication applications [28,33].

Different research groups have extensively investigated FAPbI_3_-based photodetectors. The responsivity of FAPbI_3_ perovskites typically ranges from 0.09 to 0.44 A W^−1^, with a specific detectivity between 1.8 × 10^12^ and 4.8 × 10^12^ Jones, and linear dynamic range (LDR) values reaching up to 207 dB [34,35]. It has been observed that cesium (Cs) concentration strongly influences device performance parameters, including LDR, detectivity, responsivity, and response speed. A photodetector based on FAPbI_3_ with 10% Cs incorporation exhibited a responsivity of 0.762 A W^−1^, a specific detectivity of 5 × 10^13^ Jones, a response time of 59.25/53.85 µs (rise/decay), and an LDR of 92 dB [36]. In contrast, increasing the Cs content to 15% significantly enhanced the responsivity to 5.7 A W^−1^ and achieved a specific detectivity of 2.7 × 10^13^ Jones, along with a much faster response time of 45/91 ns. However, this improvement in speed and responsivity was accompanied by a reduction in LDR to 32 dB [37]. Recently, Hamazakil et al. developed low-voltage-driven and enhanced voltage-driven diodes based on CsFAPbI_3_ by incorporating ionic liquids [38]. Amin et al. used the thermal evaporation technique to fabricate a Cs_0.06_FA_0.94_Pb(I_0.68_Br_0.32_)_3_-based photodetector. The fabricated devices exhibited excellent sensitivity to low light, capable of detecting weak light (a few pW cm^−2^), with a 1.3 µs response time and good operational stability [39]. The triple cation lead-halide perovskite photodetector exhibited a responsivity of 0.52 A/W, a specific detectivity of 8.88 × 10^12^ Jones, a high on/off ratio of 7.3 × 10^5^, and a rapid rise time of 19 μs [40]. The FA_0.7_Cs_0.3_Pb(I_0.8_Br_0.2_)_3_ flexible photodetectors exhibit 2.8 × 10^13^ Jones specific detectivity, high responsivity 1.1 A·W^–1^, and a rapid rise time [41].

In this study, we report on mixed Cs_x_FA_1−x_PbI_3_ perovskite detectors deposited through a simple spin-coating technique as an alternative to the technology-demanding Si detectors. The measured photo-response at CW excitation for CsFa detectors was compared to the response of a Si photodiode as well as perovskite-based detectors. Previous studies on Cs/FA mixed perovskite photodetectors have reported responsivities of approximately 0.48–0.49 A W^−1^ with response times in the microsecond range (1.2–2.1 µs) depending on the Cs composition [42]. Reported devices in this study demonstrated a linear dynamic range (LDR) of about 135 dB with detectivity values around 6.1 × 10^11^ Jones. In comparison, the photodetector developed in this work exhibits a responsivity of 0.28 A W^−1^ at 532 nm, which is comparable to that of commercial silicon photodiodes. More importantly, the optimized device demonstrates a significantly faster rise time of 27.5 ns, corresponding to a modulation bandwidth of 12.7 MHz, which is considerably faster than the previously reported microsecond-scale response times of Cs/FA mixed perovskite photodetectors. In addition, the device achieves a wide LDR of 129.2 dB and a specific detectivity up to 1.2 × 10^12^ Jones, indicating competitive sensitivity while offering substantially improved temporal response. These results highlight the potential of the present Cs/FA mixed perovskite photodetectors for high-speed and high-sensitivity photodetection applications. The 20% cesium alloyed detector showed the best spectral response. The temporal response of detectors after pulsed laser excitation was also investigated to reveal the fastest rise time and fall time values in the Cs_0.2_FA_0.8_PbI_3_ photodetector.

## 2. Materials and Methods

### 2.1. Essential Precursors

CsI (cesium iodide, 99.99%), PbI_2_ (lead iodide, 99.99%), and FAI (formamidinium iodide, 98%) were obtained from Lumtec (New Taipei City, Taiwan). DMSO (dimethyl sulfoxide, 99.55%) and DMF (*N*,*N*-dimethyl formamide, 99.55%) were obtained from TCI (Tokyo Chemical Industry, Tokyo, Japan).

### 2.2. Solution Synthesis and Detector Deposition

Different 1.5 M perovskite (Cs_x_FA_1−x_PbI_3_) solutions were prepared by varying the Cs concentration in the precursor solution. For this purpose, CsI, PbI_2_, and FAI were weighted with x:1-x:1 ratio to PbI_2_ and dissolved in an anhydrous DMF (900 µL) and DMSO (100 µL) mixture overnight in the nitrogen-filled glove box. The solution was filtered with a 0.22 μm filter paper before deposition. Nickel oxide precursor solution was prepared from nickel(II) nitrate hexahydrate (Ni(NO_3_)_2_·6H_2_O) in 2-methoxyethanol and acetylacetone.

Firstly, 1.1 mm thick ITO substrates 15 × 20 mm (Ossila, Solpro Business Park, Windsor Street, Sheffield S4 7WB, UK) were patterned using 355 nm, 100 Hz pulses from an Nd: YAG laser (Coherent Infinity 40–100, Santa Clara, CA, USA) and scanning on computer-controlled xy stages to obtain 0.2–6 mm^2^ detector area sizes. Then the patterned ITO substrates were cleaned with a conventional RTA process and placed under UV-ozone for 15 min at 120 °C. A one-step deposition method was used to deposit the NiO layer by a spin coater (POLOS Spin150i, Putten, The Netherlands) at 3000 rpm for 40 s (acceleration 800 rpm/s) with subsequent annealing at 300 °C for 1 h. Then they were transferred into a nitrogen-filled glove box (MBRAUN model: MB-20-G/MB-200G-W, Garching, Germany). The perovskite absorber films were deposited at 4000 rpm for 40 s (acceleration 800 rpm/s). Chlorobenzene, used as an antisolvent, was added 10 s before completing the rotations to obtain a smooth, homogeneous perovskite layer. After that, the structure was placed on a hot plate at 100 °C for 30 min to obtain a black perovskite film. The thermal evaporator (Inficon, East Syracuse, NY, USA) was used to deposit 30 nm and 60 nm C_60_ and Ag electrodes on the perovskite. Reference perovskite layers were prepared on RTA-cleaned glass substrates. At the end, the fabricated detectors (reference samples) were encapsulated with epoxy and glass for further characterization.

### 2.3. Characterization

The X-ray diffraction (XRD) spectra were obtained by a Rigaku Smart Lab diffractometer (Rigaku, Tokyo, 196-8666 Japan) with 9 kW Cu Kα rotating anode X-ray tube and a scintillation detector SC-70 (Rigaku, Tokyo, Japan). The quality of perovskite layers was characterized by scanning electron microscope (SEM, Hitachi SU8230 microscope, Hitachi High-Tech Corporation, Tokyo, Japan) in scanning and cross-section measurements.

The detector IV curves and current dependences on light intensity were determined using a Keithley 2401 (TEKTRONIX, INC., Beaverton, OR, USA) source measure unit. The intensity of the exciting 532 nm CW laser was varied using calibrated neutral density filters. The energy of the excitation light was measured by a PD300-UV (Jerusalem, Israel) power sensor. Spectral responsivities were measured with the Edinburgh Instruments spectrophotometer (FLS 980, Livingston, UK). The detector capacities C were measured using a capacity meter Mastech MS6013 (Taipei City, Taiwan). The temporal characteristics of the detectors were tested using a 527 nm pulsed Nd: YLF laser (10 ps pulse duration) and fast photocurrent detection using a 6 GHz oscilloscope (Lecroy SDA6000, Heidelberg, Germany) synchronized with the laser pulses by a photodetector (Thorlabs DET08CFC/M, Newton, NJ, USA). The PL decays of the perovskite layers and detectors were obtained using time-resolved photoluminescence (TRPL) at 532 nm excitation with a measurement setup described in a previous report [43].

## 3. Results and Discussion

**Figure 1:**
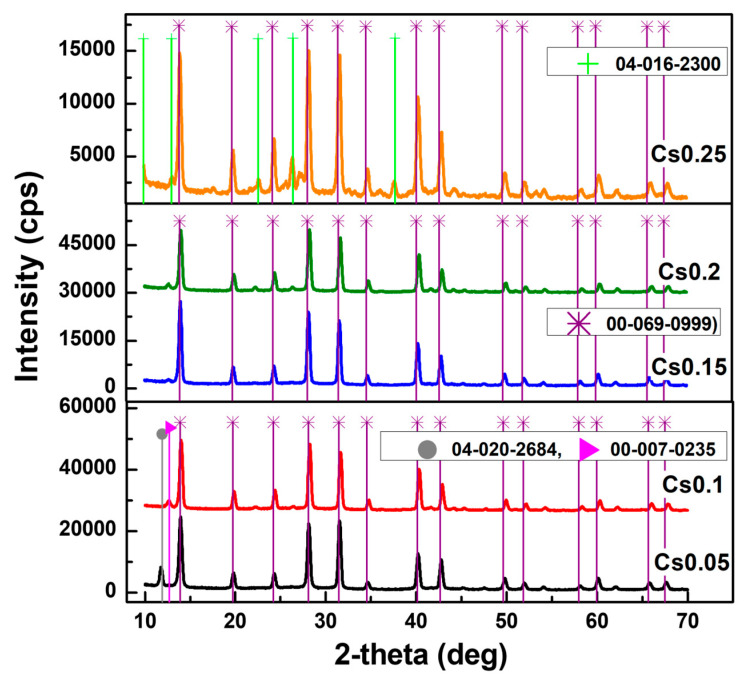
XRD patterns of perovskite layers Cs_x_FA_1−x_PbI_3_ (x = 0.05–0.25). Symbols indicate peaks according PDF cards with numbers provided on the plot. Curves are vertically shifted for clarity.

Figure 1 shows the XRD patterns of Cs_x_FA_1−x_PbI_3_ films with different Cs contents (x), together with the corresponding PDF card data. All samples mainly exhibit the cubic FAPbI_3_ phase (PDF #00-069-0999, *Pm*–3*m*), with eight characteristic diffraction peaks, consistent with previous reports [44]. Weak peaks related to the trigonal Cs_4_PbI_6_ phase (PDF #04-020-2684) appear in films with low Cs content, while the orthorhombic CsPbI_3_ phase (PDF #04-016-2300) is observed in the film with 25% Cs. A stable cubic FAPbI_3_ phase (PDF #00-069-0999) is observed in films of 10–20% Cs concentration. The transition from cubic FAPbI_3_ to orthorhombic CsPbI_3_ phase is indicated by diffraction peaks at 9.94°, 13.06°, 22.66°, 26.46°, and 37.74° in samples containing 25% Cs. A small peak at 12.67°, corresponding to PbI_2_ (PDF #00-007-0235), becomes most evident for x = 0.1–0.2. The presence of PbI_2_ suggests effective defect passivation in the perovskite films [45,46]. Additionally, the continuous shift of diffraction peaks toward higher 2θ values with increasing Cs content confirms Cs^+^ incorporation into the FAPbI_3_ lattice, resulting in lattice contraction due to the smaller ionic radius of Cs^+^ compared to FA^+^ [47].

**Figure 2:**
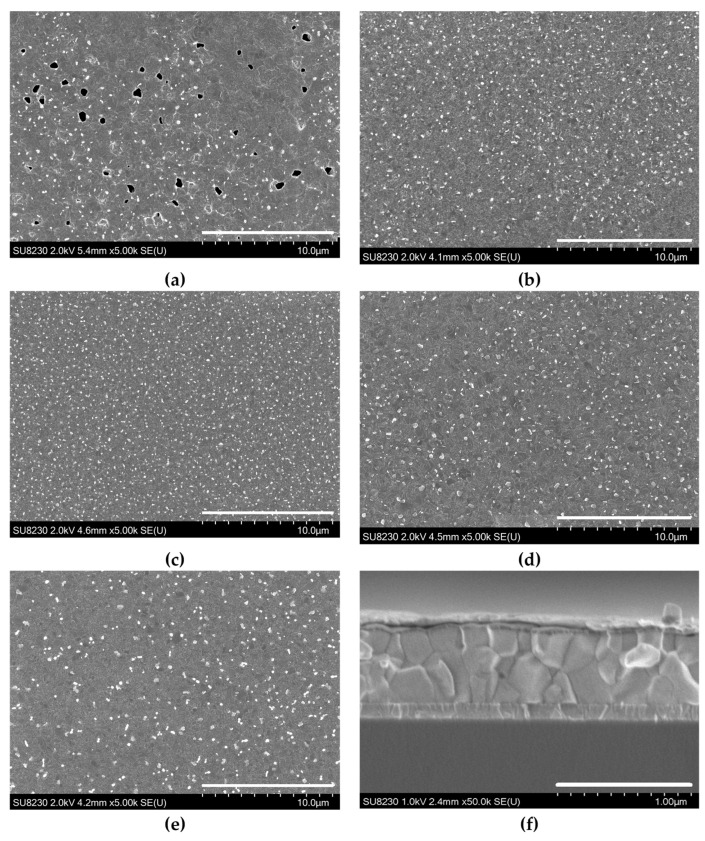
Morphology of perovskite Cs_x_FA_1−x_PbI_3_ (x = 0.05–0.25) layers (**a**–**e**) and Cs0.2 detector cross-section (**f**).

Therefore, XRD patterns of the perovskites with 10–20% Cs show the most stable lattice structure with the least structural defects. Layers with composition outside the 5–25% range showed non-cubic perovskite mixed phases being not suitable for light harvesting due to larger band gaps [48]. The crystallite quality inspected by SEM (Figure 2a–e) correlates with XRD, showing the best layers with 10–20% Cs content (5% Cs led to a high number of pinholes, which are detrimental for detector operation). The device cross-section is shown in Figure 2f.

**Figure 3:**
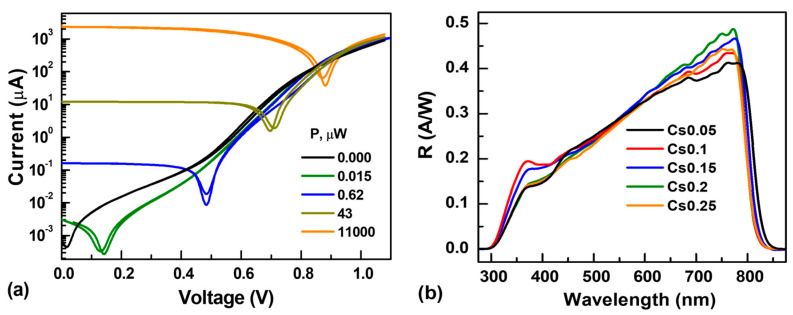
IV curves of Cs0.1 detector under dark and variable-light conditions (**a**), and spectral responses of the studied detectors (**b**).

**Figure 4:**
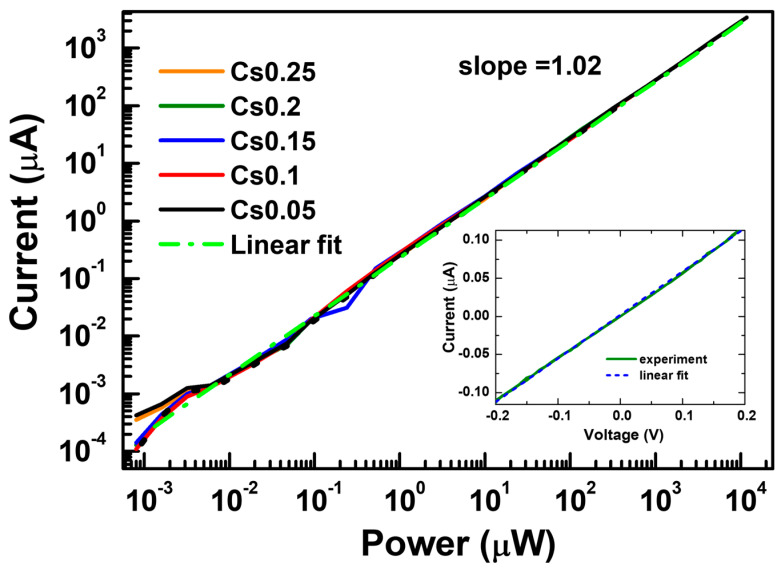
Linearity of the photo response at CW excitation for Cs_x_FA_1−x_PbI_3_ detectors. The dashed line shows a linear fit on a log–log plot, while the dotted one indicates a comparable response of a Si photodiode. The inset shows the dark IV curve on a linear scale used for the Cs0.2 detector shunt resistance determination.

The detectors were operated in photovoltaic mode without bias, which is important for low-light detection in energy-saving applications. The IV curves show almost symmetric responses in the dark conditions, while a shift of the minimum is observed under illumination (Figure 3a). The spectral response curves of the photodetectors based on the Cs_x_FA_1−x_PbI_3_ perovskites are presented in Figure 3b with a cutoff wavelength close to 834 nm. The detectors’ response is linear in a wide nW–mW excitation range (see Figure 4), proposing their applications in linear wide dynamic range sensing. In the 10–1000 mW range at 532 nm, the average detector’s photo-responsivity was 0.26–0.27 A/W (see Table 1), which is very similar to a standard Si and graphene photodiode [49,50] with 0.261 A/W, as well as comparable with the FAPbI_3_-based photodetector [34,35]. The responsivity (R) of the photodetectors shows best values for 10–20 samples. The UV edge is limited by the absorption in the glass substrate and nickel oxide. In contrast, the infrared edge is determined by the bandgap of the perovskite, which slightly increases with the Cs amount due to a lattice contraction. The observed non-monotonic shift in the bandgap with composition can be attributed to interactions between the FA cation and the inorganic lattice. Such interactions reduce rotational disorder and enhance structural stability, rather than arising from changes in the electronic band structure [42]. Photoluminescence peaks were observed at 764, 762, 776, 773, and 754 nm for Cs0.05, Cs0.1, Cs0.15, Cs0.2, and Cs0.25, respectively. The peak variation is attributed to changes in crystallinity and phase evolution in the perovskite films [51,52]. The largest blue shift is observed for Cs0.25, which is most plausibly linked to an iodide-rich phase [53] as confirmed from the XRD analysis.

To further quantitatively assess the device performance with the Cs_x_FA_1−x_PbI_3_ film, current responsivity (*R_i_*) was calculated using the following equation [40]:

(1) $$R_{i}=\;\frac{I_{\lambda }-I_{d}}{P_{\lambda }A}$$

where *I*_λ_ is the photocurrent, *I_d_* is the dark current (average *I*_d_ = 0 in photovoltaic detectors), *P*_λ_ is the incident power intensity, and *A* is the area of the device. By using the above equation, responsivity was calculated (see Table 1). The linear dynamic range (LDR) is particularly important for practical photodetection applications, especially in image sensors, since they need to operate over a broad intensity range to extract the detected light power, and the corresponding photocurrent was also calculated by the following expression on a logarithmic scale [39]:

(2) $$LDR=20log\frac{L_{max}}{L_{min}}$$

where *L_max_* and *L*_min_ are the maximum and minimum limits of the light intensity in the linear photo-response range, respectively. Figure 4 shows the current of photodetectors illuminated by a 532 nm green light at different powers. It can be clearly observed that the photocurrent gradually increases with the light power. Linear slope indicates the trap-mediated nonradiative recombination in the devices, which value can be changed with trap density [54]. The lower limit of the LDR is restricted by the light intensity of the source. We calculated the expected LDR value to be 129.2 dB based on the illumination intensity corresponding to the noise current, which agrees with that of a commercial photodetector (Si or InGaAs) [33,39,55] and larger than the 10% and 15% Cs doped in FAPbI_3_ perovskite-based photodetectors having 92 dB and 32 dB, respectively [36,37]. Such a large linear response may result from the good carrier transport and reduced trap density in the Cs_x_FA_1−x_PbI_3_ polycrystalline films [13]. Recent research provided a higher LDR = 153.61 dB in a 2D perovskite single-crystalline photodetector [56] and LDR = 160 dB in perovskite photodetectors enabled by 3D/2D/3D heterostructures unipolar barrier [57]. However, the bandwidth values were much lower: 250 Hz and 344 kHz, respectively (responsivities were 2.2 A/W and 0.44 A/W, respectively).

**Figure 5:**
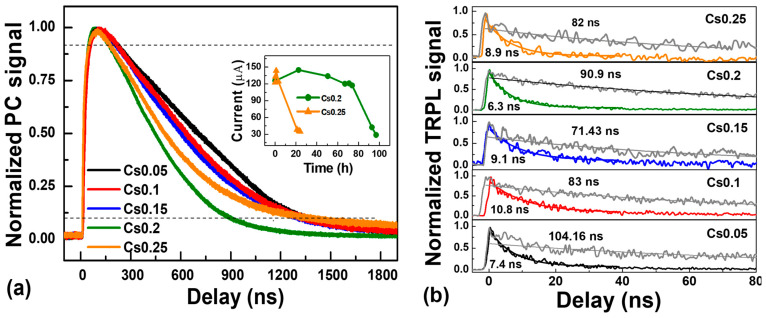
Normalized temporal response of Cs_x_FA_1−x_PbI_3_ detectors after pulsed laser excitation. Dashed lines indicate the signal levels at 10% and 90% (**a**). TRPL decays for perovskite layers (grey curves) and corresponding detectors with indicated corresponding TRPL decay times (**b**). The inset in (**a**) shows the degradation of the photo-response at 6 mW/cm^2^ white LED power.

Finally, the response speed of perovskite photodetectors was evaluated by measuring the transient photocurrent decays. In this measurement, the device was illuminated by 10 ps laser pulses, and the transient photocurrent signal was recorded after excitation. The bandwidth (*BW*) is related to detector rise time as *BW* = 0.35/*t_r_*. The rise time t_r_ and fall time t_f_ correspond to the signal change from 10% to 90% and back to 10% [13]. Under 0 V bias, the Cs_x_FA_1−x_PbI_3_ photodetectors achieved a rise time between 27–41 ns and a fall time of 700–1200 ns (Figure 5). The Cs0.2 detector showed the shortest rise and fall time, indicating its best perovskite phase purity, which is much better than in the other photodetectors working in the 300–850 nm spectral range with the same material and fast photo-response in the MHz frequency range [36,40,58]. The variation in the rise and fall time is due to the carrier transport properties and trap state densities [13]. The obtained values were averages over 12 devices; the error appears due to the fluctuations of the perovskite layer thickness. The reduction of the photocurrent at continuous illumination is shown in the Figure 5a inset. The Cs0.25 shows much faster degradation vs. Cs0.2 (10 h vs. 80 h), which can be explained by faster decomposition and cubic–orthorhombic phase transition [59,60]. For low-light detection, perovskite devices would exhibit longer lifetimes because degradation is proportional to the absorbed light dose [61].

The devices have a faster response time and shorter recovery time (fall time) mainly due to the following three factors: (1) lower number of defects, thus the smaller probability of electrons to be trapped, so in the vicinity of the interface, electrons do not undergo trapping or de-trapping, which greatly decreases the recovery time [45]; (2) the strong built-in electric field formed by hybrid heterojunctions facilitates the separation of electron–hole pairs and the transfer of charges [62], resulting in a fast photo response [46,63]; (3) material with excellent carrier transport performance [64,65,66] causes carriers to drift to the electrode more quickly [47].

**Figure 6:**
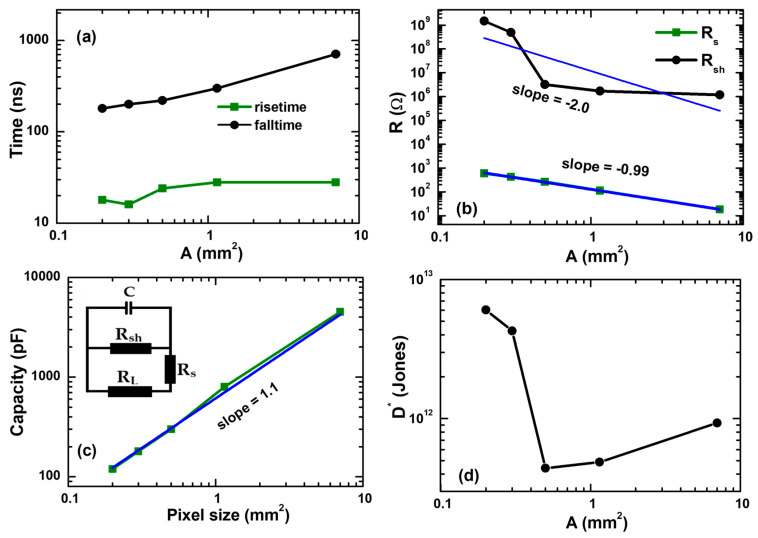
Capacity *C* (**a**), series *R_s_* and shunt resistances *R_sh_* (**b**), rise time and fall time (**c**), noise current and detectivity (**d**) dependences on device area *A* for 20% Cs composition. Inset (**c**) shows the detector electrical model.

In our study, the rise times of the detectors were determined by the speed of electron and hole separation by the contacts [67]. This is confirmed by measuring PL decay times of pristine perovskite layers on glass and corresponding detector structures (Figure 5b). Pristine perovskite layers show exponential PL decay times of ~80–100 ns, which correspond to a 140–200 ns carrier lifetime (a 2× multiplier appears because PL is proportional to Δ*N*^2^ [43], where Δ*N* is the photo-excited carrier density). In the detectors, the PL decay times become much shorter and reach 6 ns, showing effective charge separation. The corresponding exponential carrier extraction time is twice as large (12 ns, see values in Table 1). Fall times of the PL decays are 2.2 times slower than the exponential decay times [67]. Thus, the calculated carrier extraction fall time of 27.7 ns in the Cs0.2 detector well coincides with the current rise time of the detector. The faster carrier extraction in the 0.2 detector indicates a larger mobility for the latter perovskite composition, as the carrier lifetimes are similar in all samples, indicating a similar defect density. Rise times weakly depend on the detector area, as observed in Figure 6a. That is a consequence of the detector device’s area-independent charge separation processes. Shorter rise times in the smallest detectors may indicate more efficient charge separation (due to a larger built-in electric field) in the small detectors as a presumable consequence of the absence of pinholes, as evidenced by strongly increased shunt resistances (see Figure 6b).

The fall time of the detector response is determined by the detector capacity *C* (see Figure 6c), discharge through the parallel series + load (*R_s_ + R_L_*), and shunt *R_sh_* resistances (see detector scheme in Figure 6c inset; *R_L_* = 50 Ω). The *C* values correspond to the perovskite dielectric constant ε = 36 ± 3 as obtained using a standard *C* = εε_0_*A*/*d* formula (ε_0_ is the static dielectric constant, *d* = 500 nm is the perovskite thickness); a similar value of 35 was previously reported [68]. The corresponding exponential *RC* discharge time is τ_d_ = *C*/(1/(*R_s_* + *R_L_*) + 1/*R_sh_*). It is related to fall time as *t_f_* = 2.2 τ_d_. Using the experimental capacity of the detectors and fall time values (see Figure 6), the series resistances were calculated (Figure 6b). They are linearly decreasing with the device area, while the capacity linearly increases. The shunt resistances were determined using the dark IV curves (see Figure 4 inset), where the slopes correspond to 1/(*R_sh_* + *R_s_*).

The specific detectivity of photovoltaic detectors can be evaluated using the equation for noise equivalent power NEP and dark current *I_dark_*:

(3) $$D^{*}=\frac{{\left(A\cdot BW\right)}^{1/2}}{NEP}$$

(4) $$NEP=\frac{I_{dark}}{R_{i}}$$

Here *e* is the elementary charge, dark current is determined by the thermal noise *I_dark_* = (4*k_B_T*·*BW*/*R_sh_*)^1/2^ [69]. Shunt resistance (*R_sh_*) is calculated at V = 0 V. The unit of detectivity is 1 Jones = 1 cm·Hz^1/2^/W. Figure 6d shows the variation of D* vs. *A*, providing values in 7 × 10^11^–7 × 10^12^ Jones range. Shunting pinholes plausibly are determining this variation. The D* values for different composition detectors are provided in Table 1. Recent studies have reported significant improvements in the performance of perovskite-based photodetectors. By varying the Cs content in FA-based perovskites, detectors based on Cs-doped FAPbI_3_ achieved detectivity values in the range of 10^7^–10^11^ Jones [70], along with an LDR of between 32 and 135 dB [35,42]. Similarly, FAPbI_3_ quantum dot/graphene photodetectors demonstrated a detectivity of 1.89 × 10^10^ Jones under 365 nm light irradiation [70]. In comparison, the α-CsPbI_3_/ZnO photodetector exhibited superior performance, delivering a photoresponsivity of approximately 8.2 A W^−1^, an ON/OFF ratio of about 2.4 × 10^4^, and a specific detectivity of approximately 1.4 × 10^12^ Jones [71]. For n-Si/MAPbBr_3_ quantum dot-based detectors, an exceptionally high responsivity of 10^7^ A W^−1^ was achieved due to an ultrahigh gain of 10^8^, resulting in a detectivity of approximately 10^12^ Jones [72]. Furthermore, Amin et al. reported that Cs_0.06_FA_0.94_Pb(I_0.68_Br_0.32_)_3_-based photodetectors achieved a specific detectivity of 7.50 × 10^12^ Jones with a responsivity of 0.360 A W^−1^ [39]. Likewise, Zhang et al. fabricated a photodiode with the structure TiO_2_/FAPbI_3_/Spiro-OMeTAD/Ag single crystals, which demonstrated a responsivity of 0.95 A W^−1^ and a specific detectivity of 2.8 × 10^12^ Jones [34]. In this work (see Table 1), the fabricated photodetectors demonstrate performance comparable to several commercial photodiodes and previously reported perovskite-based devices in terms of responsivity, highlighting its competitive photo-detection capability and good sensitivity for practical applications.

## 4. Conclusions

Cs_x_FA_1−x_PbI_3_ perovskite photodetectors with Cs contents ranging from x = 0.05 to 0.25 were developed and evaluated for visible-light detection. Structural characterization confirmed that compositions with x = 0.1–0.2 form stable cubic lattices and uniform, black, defect-free films. Devices fabricated in a vertical p–i–n architecture exhibit linear photocurrent response over a wide optical power range (1 nW–10 mW), leading to a 129.2 dB linear dynamic range and a photoresponsivity of 0.28 A/W at 532 nm. Temporal response of the detectors after pulsed laser excitation reveals that the Cs_0.2_FA_0.8_PbI_3_ composition provides the fastest response, with a rise time of 27.5 ns, corresponding to a modulation bandwidth of 12.7 MHz. Reduction of the series resistance would further enhance the bandwidth. These results demonstrate that Cs_0.2_FA_0.8_PbI_3_ is a promising perovskite composition for high-speed, cost-effective photodetectors suitable for VIS range imagers and light communication applications.

## Figures and Tables

**Table 1:** Electrical parameters of the detectors.

Detector	*R_i_* @ 532 nm (A/W)	Peak Wavelength (nm)	Rise Time (ns)	Fall Time (ns)	Bandwidth (MHz)	*I_th_* (nA)	D* 10^12^ (Jones)	Layer τ_R_ = 2τ_PL_ (ns)	Detector τ_extr_ = 2τ_PL_ (ns)
Cs0.05	0.271	764	35.6 ± 5.4	1098 ± 192	9.8 ± 1.5	18	0.02	14.8	208
Cs0.1	0.263	762	41.7 ± 4.5	1105 ± 163	8.4 ± 0.9	0.25	1.48	21.6	166
Cs0.15	0.275	776	34.5 ± 3.5	1046 ± 105	10.1 ± 1.1	0.31	1.2	18.5	143
Cs0.2	0.272	773	27.5 ± 2.8	708 ± 71	12.7 ± 1.2	0.46	1.0	12.6	182
Cs0.25	0.262	754	33.7 ± 4.1	1166 ± 112	10.4 ± 1.3	0.59	0.65	17.8	164

*I_th_* is the thermal noise current (RMS), τ_PL_ is the PL decay time, τ_R_ is the recombination time, τ_extr_ is the carrier extraction time.

## Data Availability

The original contributions presented in this study are included in the article. Further inquiries can be directed to the corresponding authors.

## Abbreviations

The following abbreviations are used in this manuscript:

CaTiO_3_	Calcium titanate
XRD	X-ray diffraction
SEM	Scanning electron microscope
EQE	External quantum efficiency
CsI	Cesium iodide
PbI_2_	Lead iodide
FAI	Formamidinium iodide
DMSO	Dimethyl sulfoxide
DMF	*N*,*N*-dimethyl formamide
LDR	Linear dynamic range
